# Shape- and Size-Controlled Synthesis of Silver Nanoparticles Using *Aloe vera* Plant Extract and Their Antimicrobial Activity

**DOI:** 10.1186/s11671-016-1725-x

**Published:** 2016-11-25

**Authors:** Kaliyaperumal Logaranjan, Anasdass Jaculin Raiza, Subash C. B. Gopinath, Yeng Chen, Kannaiyan Pandian

**Affiliations:** 1 Department of Inorganic Chemistry, University of Madras, Guindy Campus, Chennai, 600025 Tamil Nadu India; 2 Institute of Nano Electronic Engineering, University Malaysia Perlis, Kangar, 01000 Perlis Malaysia; 3 School of Bioprocess Engineering, University Malaysia Perlis, Arau, 02600 Perlis Malaysia; 4 Department of Oral and Craniofacial Sciences, University of Malaya, 50603 Kuala Lumpur, Malaysia; 5 Oral Cancer Research and Coordinating Center (OCRCC), Faculty of Dentistry, University of Malaya, 50603 Kuala Lumpur, Malaysia

**Keywords:** *Aloe vera* gel, Silver nanoparticles, Microwave synthesis, Antimicrobial activity

## Abstract

**Abstract:**

Biogenic synthesis of silver nanoparticles (AgNP) was performed at room temperature using *Aloe vera* plant extract in the presence of ammoniacal silver nitrate as a metal salt precursor. The formation of AgNP was monitored by UV-visible spectroscopy at different time intervals. The shape and size of the synthesized particle were visualized by scanning electron microscopy (SEM) and transmission electron microscopy (TEM) observations. These results were confirmed by X-ray powder diffraction (XRD) and Fourier transform infrared spectroscopy (FTIR) analyses and further supported by surface-enhanced Raman spectroscopy/Raman scattering (SERS) study. UV-visible spectrum has shown a sharp peak at 420 nm and further evidenced by FTIR peak profile (at 1587.6, 1386.4, and 1076 cm^−1^ with corresponding compounds). The main band position with SERS was noticed at 1594 cm^−1^ (C–C stretching vibration). When samples were heated under microwave radiation, AgNP with octahedron shapes with 5–50 nm were found and this method can be one of the easier ways to synthesis anisotropic AgNP, in which the plant extract plays a vital role to regulate the size and shape of the nanoparticles. Enhanced antibacterial effects (two- to fourfold) were observed in the case of *Aloe vera* plant protected AgNP than the routinely synthesized antibiotic drugs.

**Graphical Abstract:**

Shape and size-controlled synthesis of silver nanoparticles using *Aloe vera* plant extract
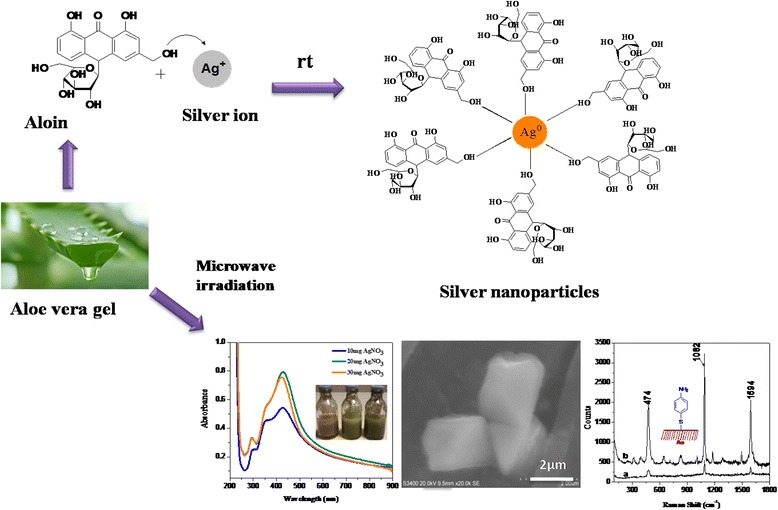

**Electronic supplementary material:**

The online version of this article (doi:10.1186/s11671-016-1725-x) contains supplementary material, which is available to authorized users.

## Background

Currently, nanoparticle-mediated studies have been predominantly demonstrated and penetrated into the interdisciplinary sciences with nanotechnology due to their potential applications [1–9]. The optical, electronic, catalytic, and electrochemical properties of the nanoparticles are undoubtedly dependent on their size, shape, and reaction medium. It can be synthesized by different methods including wet chemical method, polyol method, microwave synthesis, photochemical approach, and biogenic synthesis, the popular methods in recent days [10]. Biogenic synthesis of nanoparticles is also known to be the green chemistry method using plant origin, edible items, algae, and herbal products [11–14]. The synthesis of silver nanoparticles (AgNP) with different sizes and shapes has been considered in the past with special interest in various fields of research due to their potential to develop for novel biosensors, chemical sensors, electro-optical devices, data storage media, surface-enhanced Raman spectral spectroscopy, and biological imaging [15–19]. AgNP have pronounced to have antimicrobial properties and performed well than other metal nanoparticles [20–25].

Recently, many researchers have been using plant or herbal extracts like neem [26], *Aloe vera* [27], *Phyllanthus* [28], turmeric [29], seaweeds [30], coffee, and tea [31] as reducing agents for the synthesis of AgNP. These extracts are not only acting as a reducing agent in the synthesis of nanoparticles but also as a stabilizing agent. In the present investigation, we exploited the usage of *Aloe vera* plant extract as a reducing agent to synthesize AgNP because of its inherent antimicrobial activity against various microorganisms and gelation properties [32–35]. It is well understood that *Aloe vera* plant extract can be used to cure various diseases with their biologically important molecules. Researchers have been shown to isolate the bioactive molecules from the *Aloe vera* plant extract using high-performance liquid chromatography (HPLC) and gas chromatography–mass spectrometry (GC-MS) [36].

Microwave heating is a fast emerging and widely followed new processing technology for a variety of nanostructured materials with smaller size, narrower size distribution, and spherical, octahedron, rod, and tetrahedron shape [37–42]. In the present study, we utilized the *Aloe vera* plant extract for the synthesis of AgNP under different conditions. The basic needs of this study are to control the size and shape of AgNP as information on this aspect is very limited. Here, we have shown how the plant extract direct the size and shapes during the growth of AgNP with *Aloe vera* plant extract under microwave heating and study its catalytic efficiency in antimicrobial activity.

## Methods

### Materials

*Aloe vera* was collected from Chennai city (Tamilnadu, India). Silver nitrate (AgNO_3_), ammonia solution (25 %), and sodium hydroxide (NaOH) were purchased from SRL chemical, India. The filter discs and nutrient agar were purchased from Merck, Mumbai, India. All the preparations were done by using deionized water. For microbial studies, the sterilized water was used.

### Preparation of *Aloe vera* Plant Extract

A fully grown *Aloe vera* leaf was taken after washing thoroughly with water and removing the green skin and cutting them into small pieces, and finally, *Aloe vera* gel was ground to form a colloidal solution. The above colloidal solution was filtered and this solution was used for further analysis.

### Synthesis of AgNP

About 5 ml of 10 mM AgNO_3_ solution was taken into a 50-ml flask containing about 5 ml *Aloe vera* plant extract and finally diluted with 1 % ammonia solution to adjust the pH of the medium. The reaction mixture was allowed to stand at room temperature and the appearance of orange yellow color indicated the formation of spherical shaped AgNP.

### Synthesis of Ag Octahedron Under Microwave Irradiation

About 20 mg of AgNO_3_ was discharged into a 10 ml beaker containing different concentrations of *Aloe vera* plant extract, and 0.5 ml of 25 % ammonia solution was added to adjust the pH of the medium. Here, we have chosen three different concentrations of silver nitrate for the synthesis of Ag–Oh nanoparticles. These solutions were kept in microwave oven for a period of 60 s with an appropriate power supply, and the reaction mixture was allowed to cool until reaching the room temperature. Again, the reaction mixture was heated further for another 60 s. These heating and cooling steps were repeated three or four times to obtain a uniform-sized octahedral-shaped AgNP. Because of the intermittent heating and cooling processes, the initially formed spherical-shaped nanoparticles act as seeds for further growth of the octahedral-shaped AgNP. In this nucleation process *Aloe vera* gel plays an important role to regulate size and shape of the AgNP.

### Antimicrobial Activity

The synthesized AgNP was tested for antibacterial activity using agar disc diffusion assay method as per Clinical Laboratory Standards Institute (CLSI). Briefly, 0.5 McFarland of inoculums were made using the overnight grown bacterial strains. Strains of microorganism used in this study were gram positive and gram negative bacteria. They were diluted as 1:10 to get final inoculums of 1 × 10^7^ colony forming unit per milliliter (CFU/ml) and inoculated on the Mueller-Hinton agar (BD Difco) medium. Sterile discs of 6 mm width were dipped into the extract, AgNP solution, and extract stabilized AgNP as well as the assay control antibiotics. Discs were placed over the incubated plates followed by incubation overnight at 37 °C. The antibacterial activity was assigned by measuring the zone of inhibition (mm) formed around the disc.

### Optical, Structural, and Imaging Studies

The biogenic synthesis of AgNP was monitored by a UV-visible spectrophotometer (Shimadzu, UV-1800, Japan). The progress of the reaction and the appearance of plasmon band were monitored at different time intervals. AgNP was dried and ground (with KBR pellets) and analyzed by Fourier transform infrared spectroscopy (FTIR; Perkin Elmer Spectrum 65). All spectra were nullified against the background spectrum obtained with KBr. The X-ray powder diffraction (XRD) pattern (Bruker D8 Advance) was recorded by using Cu-Kα_1_ radiation (λ of 1.5406 Å) and nickel monochromator filtering wave tube voltage (40 kV) and tube current (30 mA). The scanning was done in the region of 2θ (from 0^o^ to 80^o^) at 0.02^o^/min. The size and shape of the AgNP was analyzed by transmission electron microscopy (TEM), and the images were obtained by JEM-1200EX instrument operated at accelerating voltage (120 kV). The particle size was analyzed by scanning electron microscopy (SEM) (FEI Quanta FEG 200). Size and shape-controlled nanoparticles were studied in a microwave oven (National model 64 N.N-GD 576 M). The sample was subjected to several short bursts of microwave irradiation at the desired frequency (2.45 GHz), at a power output of ~100 W in a cyclic mode (on 5 s, off 5 s) to avoid overheating and metal aggregation. The irradiation process was conducted as a minimum of five and maximum of 15 cycles. Surface-enhanced Raman spectroscopy/Raman scattering (SERS) was performed by Real-Time Analyzers, Inc. that incorporates a high-performance 785 nm spectrometer with RTA’s patented SERS vials for the detection of trace chemicals.

## Results and Discussion

**Fig. 1:**
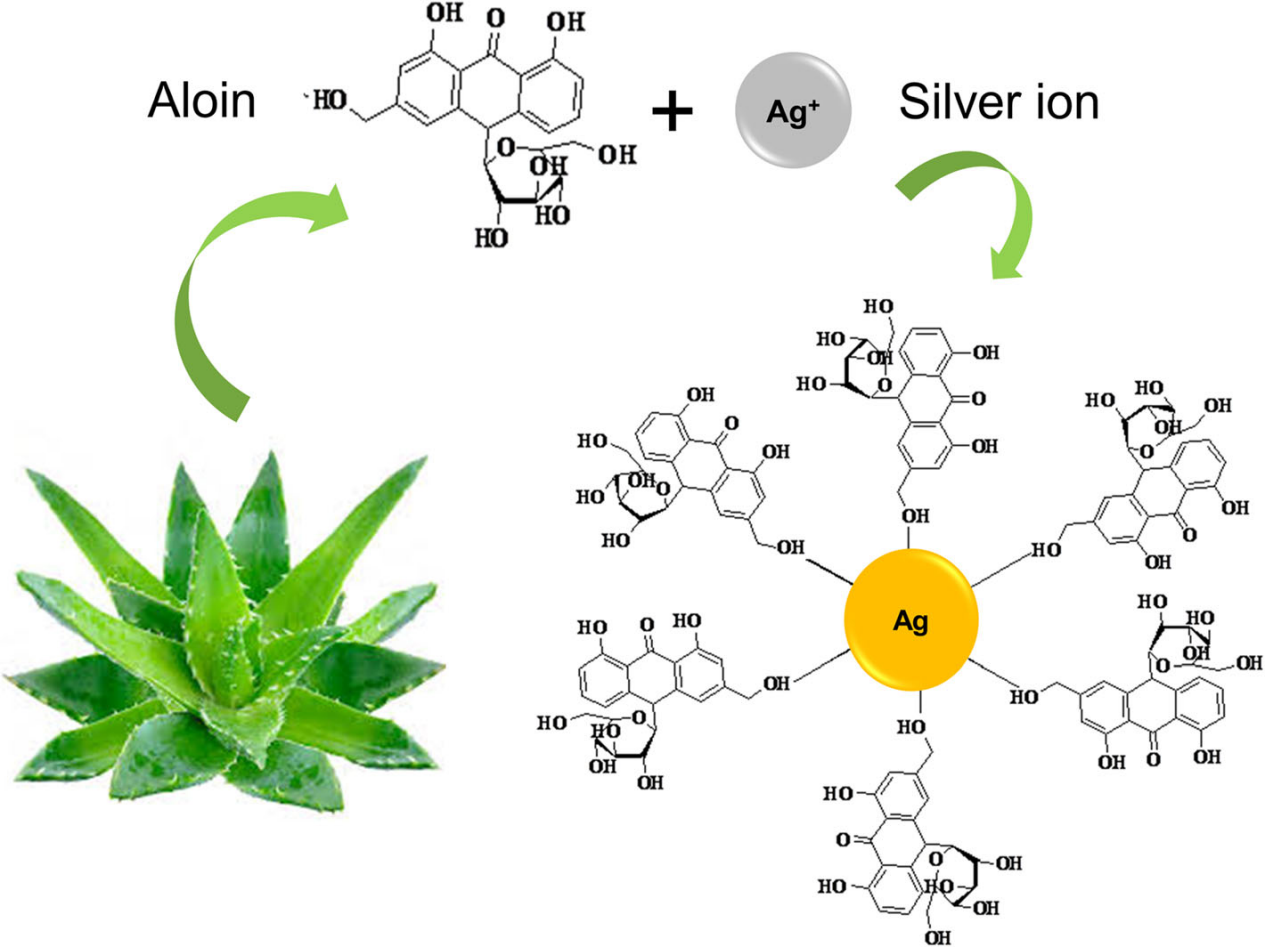
Formation of AgNP. The chemistry involved in the synthesis of AgNP using *Aloe vera* is shown

The synthesis of nanoparticles benefits for the development of clean, non-toxic and environmentally acceptable “green chemistry procedures” and involves a wide range of materials including plant sources [10–14]. Among different nanoparticles that have been proposed, AgNP was found to be with significant applications [24, 32, 34]. Different groups have been involved in synthesizing various sizes and shapes of nanoparticles under microwave condition using high boiling solvents [40]. Here, we report size and shape-controlled synthesis of AgNP using *Aloe vera* plant extract wherein the *Aloe vera* gel plays a vital role as a shape-directing agent for the growth of AgNP under microwave irradiation. The chemistry involved in the formation of AgNP is displayed in Fig. 1. For the initial assessments, we have generated AgNP under room temperature and evaluated by UV-Visible spectroscopy.

### UV-Visible Spectroscopic Analysis

**Fig. 2:**
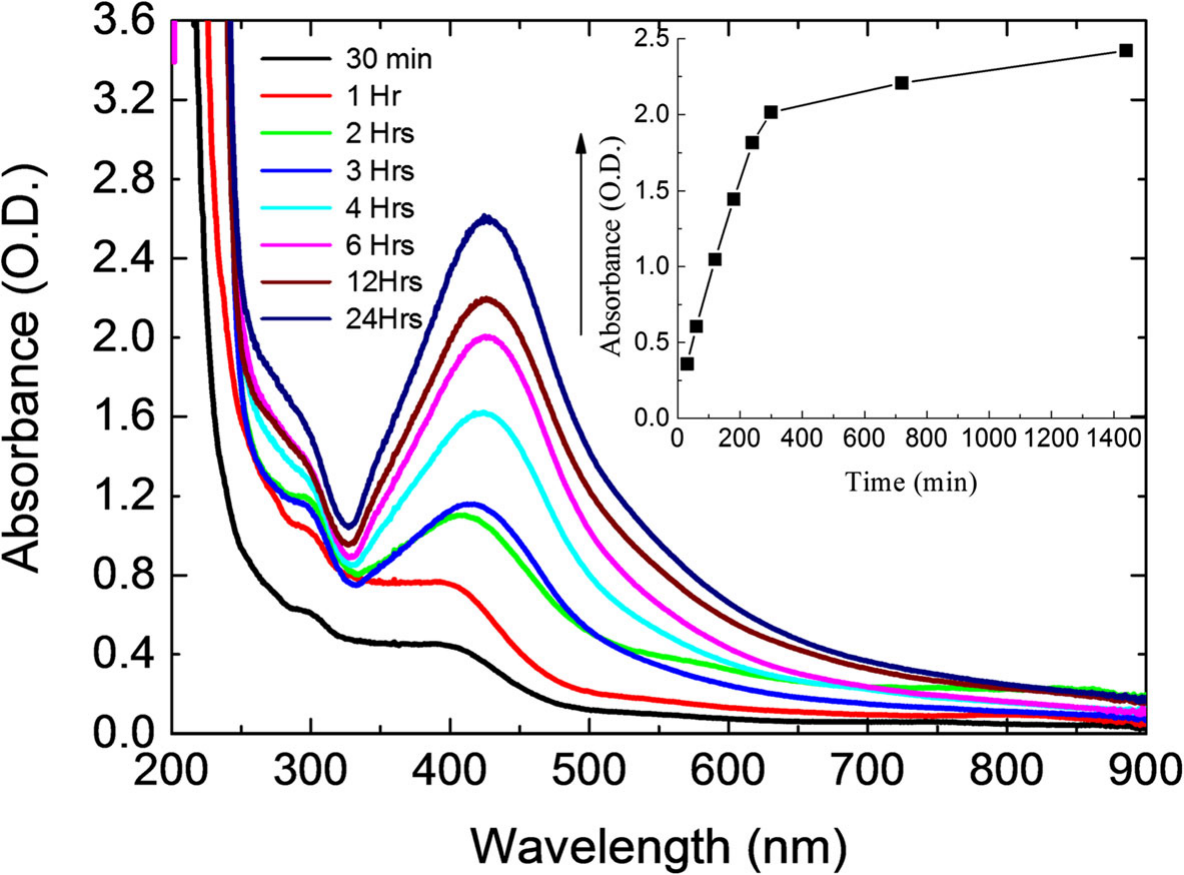
UV-visible spectra for continuous growth of AgNP. Experiments were performed in room temperature. Measurements were taken from 30 min to 24 h with different intervals. The *arrow* indicates the direction of changes with the spectra. The figure *inset* displayed the stabilization of AgNP synthesis with a given time

Figure 2 shows the UV-Visible spectra of AgNP grown with different time intervals at room temperature in alkaline pH medium. The progress of the reaction is facile when ammoniacal silver nitrate was used as a starting material in the presence of freshly collected *Aloe vera* gel, and here it was proved that the reaction is faster in alkaline pH medium. A sharp peak increment in absorbance value was noted during the addition of silver nitrate solution, and then after reaching the maximum absorbance, the reaction slowed down due to the complete reduction of Ag^+^ to Ag (0). The plasmon peak appeared at 420 nm, which indicates the formation of AgNP. After a few hours, the attainment of peak becomes stabilized (Fig. 2; inset). No obvious change was noticed in 3 h due to saturation in 2 h. However, as the duration of reaction increases, more amounts of AgNPs were formed. In order to optimize the experiment, the kinetics of the particle growth was investigated. Based on the preliminary preparation and assessment at room temperature, further synthesis was preceded by microwave heating.

### Microwave Synthesis of AgNP

**Fig. 3:**
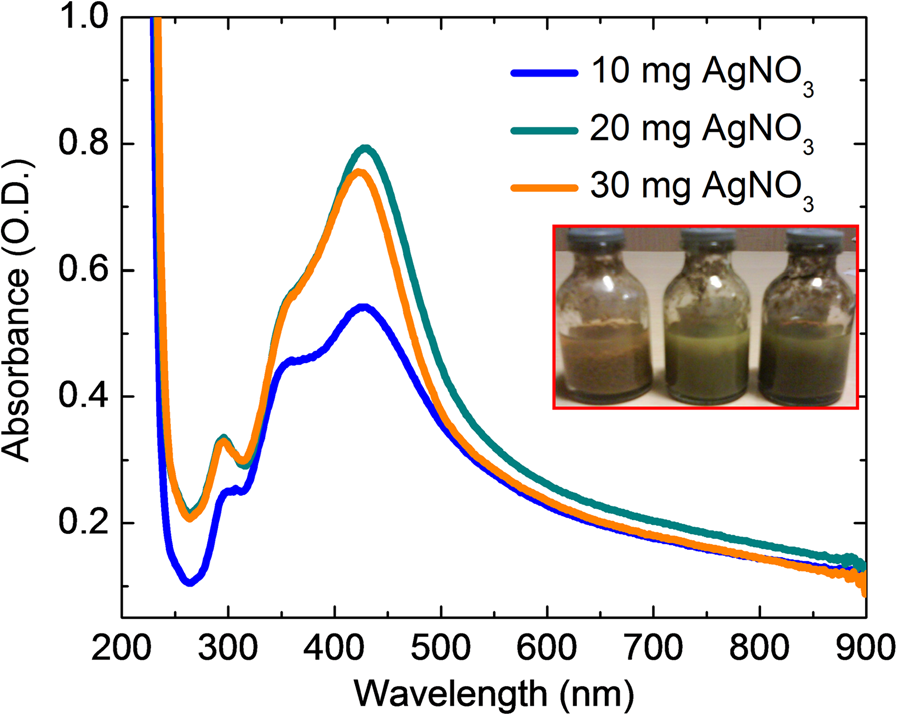
UV-visible spectra of Ag octahedron grown by microwave heating using *Aloe vera* plant extract. Different concentrations of AgNO_3_ (10, 20, and 30 mg) with MWI 750 watt, 60 s. The synthesized particles are shown in the figure *inset*

Polyol method is routinely followed to synthesize various sizes and shapes of AgNP using polyvinylpyrrolidone (PVP) as a shape-directing agent. Here, we reported that the *Aloe vera* plant extract can be used as a size and shape-directing agent for the preparation of octahedron-shaped AgNP. The addition of silver nitrate solution into *Aloe vera* plant extract yielded a spherical shaped AgNP, whereas the reaction mixture subjected to heat under microwave results in the formation of octahedron-shaped AgNP. In order to avoid the fast evaporation of solvent, the reaction mixture was kept in a microwave oven for a period of 1 min and allowed to cool, and the heating and cooling cycles were continued three to four times. The color change during the microwave heating at different intervals was monitored by UV-Visible spectral studies. The UV-Visible absorption spectrum of the octahedral-shaped AgNP is shown in Fig. 3. From the figure, three major shoulder bands were observed at 300, 350, and 430 nm ranges. The appearance of three major shoulder peaks clearly indicates the multiple electromagnetic interactions with the octahedral-shaped AgNP. This preparation was also carried out with different amounts of AgNO_3_ and shown in Fig. 3; the profile of the spectra was increased with the increment of AgNO_3_, which indicates the concentration-dependent synthesis of AgNP. At room temperature, the SPR value was higher than that obtained in the case of microwave-assisted synthesis. It is observed that the SPR band shifts to a longer wavelength with increasing particle size. The main attraction of microwave synthesis is that it yields small, uniform-sized nanoparticles in much lesser reaction time. The speedy consumption of starting materials reduces the formation of agglomerates in microwave-assisted methods and provides nanoparticles with narrow size distribution.

### Nanoscale Imaging

**Fig. 4:**
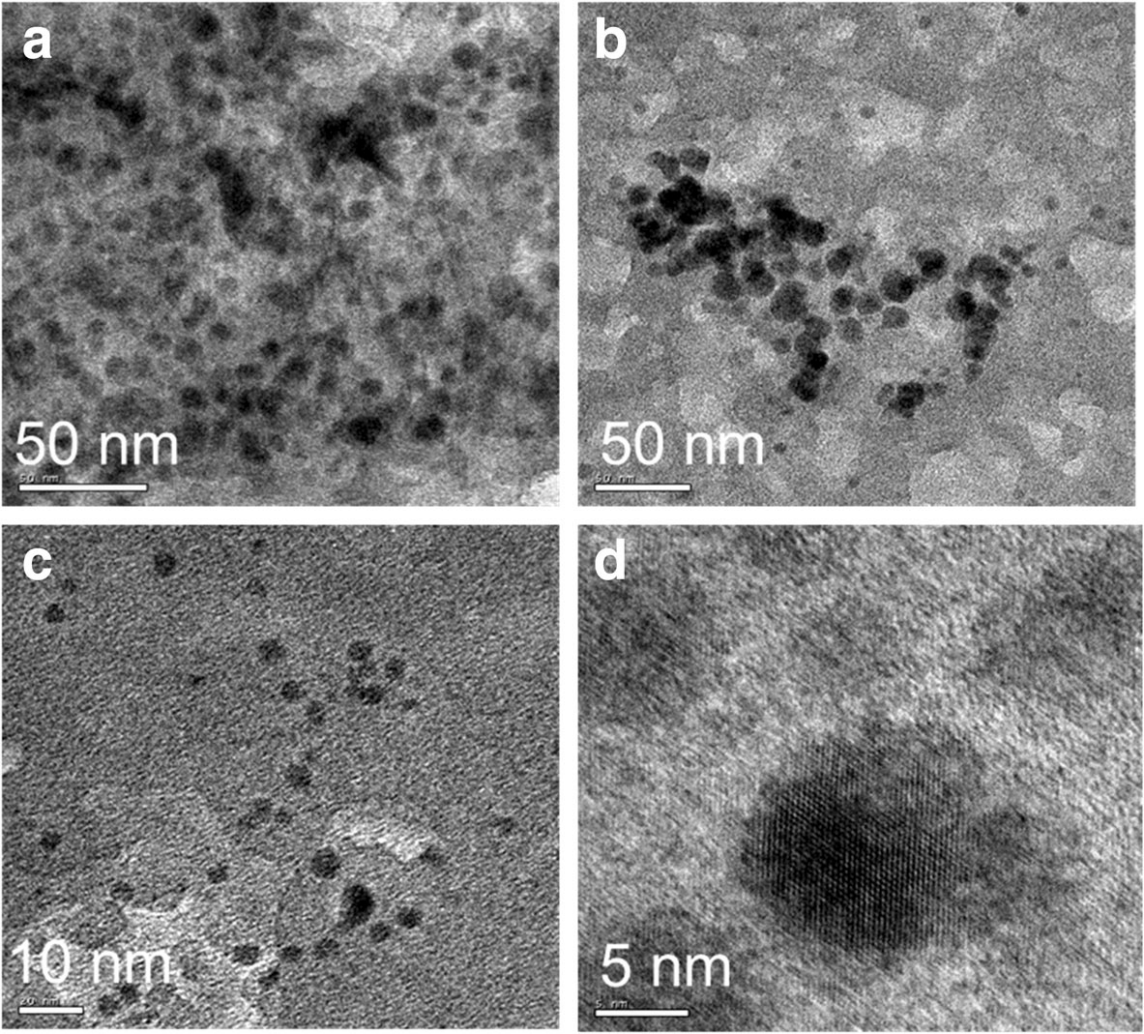
SEM image of Ag octahedral grown under microwave heating. **a**, **b**, **c**, and **d** indicates the images captured under different magnifications. *Scalebars* are shown

**Fig. 5:**
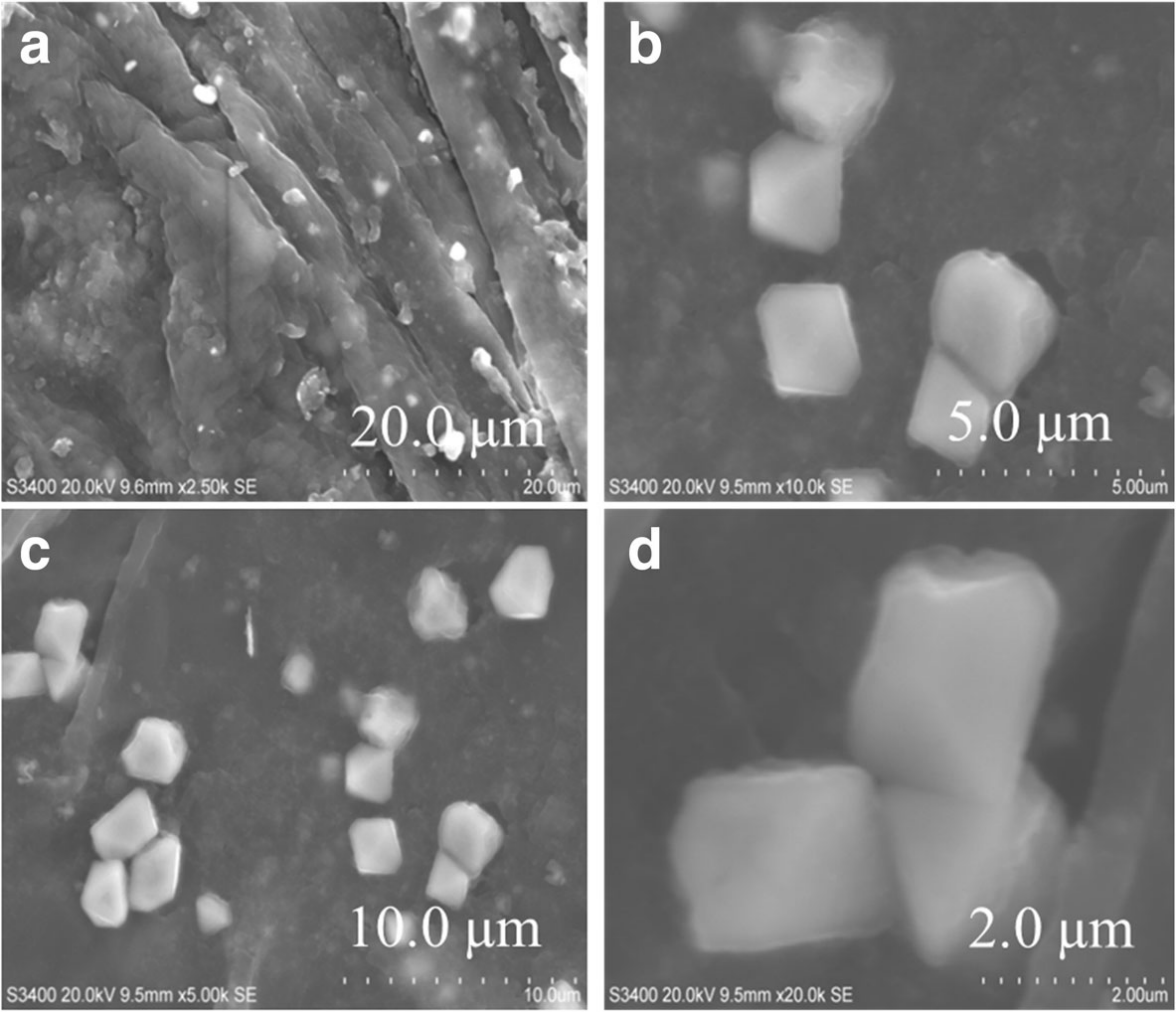
TEM images of AgNP grown using *Aloe vera* plant extract. **a**, **b**, **c**, and **d** indicates the images captured under different magnifications. *Scalebars* are shown

The obtained particles were observed under field-emission scanning electron microscopy (FE-SEM). Based on this observation, the surface morphology and the shape of AgNP were further confirmed from the resulting images with the octahedral-shaped AgNP (Fig. 4). These particles have narrow sizes and are distributed with the average size of 5–50 nm. Further, for confirmation and to support the FE-SEM results, we also observed the images of AgNP by TEM, and a clear image of AgNP was visualized (Fig. 5).

### Structural Analyses

**Fig. 6:**
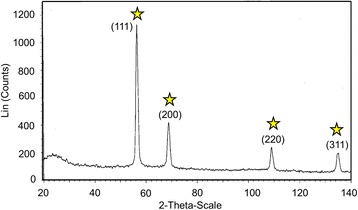
XRD spectrum of plant extract stabilized AgNP. Peaks are appeared at 111, 200, 220, and 311. Peaks are indicated by *stars*

**Fig. 7:**
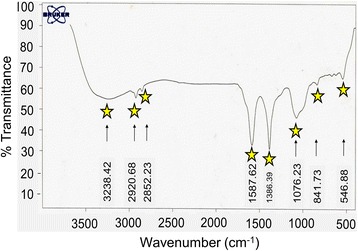
FTIR spectrum of AgNP grown using *Aloe vera* plant extract. Peak positions are shown and indicated by *stars*

For structural analyses, XRD and FTIR studies were carried out. The XRD pattern for the *Aloe vera* plant extract in the presence of AgNP is shown in Fig. 6. The XRD peaks were indexed and represented the fcc pattern of AgNP. The displayed peak indicates the nanocrystalline shaped silver particles. The FTIR spectrum of AgNP protected with *Aloe vera* plant extract is shown in Fig. 7. The observed peaks indicate the occurrence of flavanones and terpenoids that are plenty in *Aloe vera* plant extract [43]. The peaks were observed with AgNP at 1587.6 cm^−1^ (C=C groups or from aromatic rings), 1386.4 cm^−1^ (geminal methyls), and 1076 cm^−1^ (ether linkages) suggest the presence of flavanones or terpenoids on the surface of synthesized AgNP.

### Surface-Enhanced Raman Scattering (SERS) Study

**Fig. 8:**
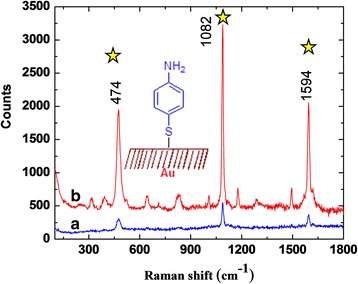
SERS studies of AgNP using *Aloe vera* plant extract. Peaks appeared at 474, 1082, and 1594. Peaks are indicated by *stars*

When molecules are on nanostructured silver or gold surfaces, they can experience the enhancement of Raman scattering, the phenomenon known as SERS. SERS-active substrates include electrochemically roughened metals, suspensions of metal nanoparticles or nanoparticles adsorbed on the surfaces, lithographically produced metal nanostructures, and vacuum-deposited metal island films. On the other hand, nanoparticles with different shapes and sizes have also shown an enhancement factor that is one of the important factors for the sensing and imaging applications [43–46]. The SERS data for the 4-aminothiophenol modified octahedral shaped AgNP are shown in Fig. 8. The appearance of the strong intense peak is due to the interaction between the electromagnetic radiations of AgNP with the probe molecules. The SERS spectrum of AgNPs displaying a group of vibrational bands were at 1594, 1082, and 474 cm^−1^. The main position band was at 1594 cm^−1^, corresponding to the C–C stretching vibration and the peaks were at 1082 cm^-1^, attributed to the C–H stretching vibration, whereas weak peak was appearing at 474 cm^−1^, corresponding to the N–H bending vibration, CNC symmetric stretching, and CO bending vibration.

To study the SERS ability of octahedral-shaped AgNP modified substrate, the SERS enhancement factor (EF) for 4-aminothiophenol (4-ATP) was estimated according to the following formula:

1 $$ \mathsf{E}\mathsf{F} = \left({\mathrm{I}}_{\mathsf{SERS}}/{\mathrm{I}}_{\mathsf{bulk}}\right)\ \mathit{\cdotp}\ \left({\mathrm{N}}_{\mathsf{bulk}}/{\mathrm{N}}_{\mathsf{surf}}\right), $$

where I_SERS_ and I_bulk_ are the vibration intensities in the SERS and normal Raman spectra of 4-ATP, respectively. N_surf_ and N_bulk_ are the number of molecules for SERS, and the number of molecules for the bulk sample under laser illumination, respectively. The N_surf_ and N_bulk_ values can be calculated on the basis of the estimated concentration of the surface species or bulk sample and the sample areas. In our experiment, 5 μl of 1 × 10^−9^ M 4-ATP solution was pipetted on the Ag–Oh NPs modified substrate, and after the droplet evaporated in air, the Raman spectrum was recorded. The resulting enhanced Raman spectrum is shown in Fig. 8. The spectral pattern is assigned for the self-assembly of 4-ATP on the surface of the octahedral AgNP. Other than major peaks, we can also see some less intense peaks which are due to Raman spectra data of the biopolymers and some of the small molecules present along the Ag–Oh NPs.

The main idea to study the EF is how the molecular signal is enhancing and how many folds. 4-aminothiophenol, 4-mercaptopyridine, and crystal violet were used as probes to study the enhancement factor. We used these compounds in SERS study to control the particle shape and size and to accurately tune nanoparticles for a specific localized surface plasmon resonance peak for optimized EF. Shape also influences SERS enhancement due to locations of high curvature such as sharp corners or tips. These locations produce unusually large electromagnetic enhancement referred as lightning rod effect; the electric field induced at the tip will be much stronger than other areas on the surface. The influence of the lightning rod effect can be observed near high curvature points on different shapes.

### Antimicrobial Studies

Since *Aloe vera* plant extract has been widely used to treat various diseases, we are interested to test its antimicrobial activity against different microorganisms. The antibacterial activity of *Aloe vera* plant extract with AgNP was compared to that of AgNP-free extract and EDTA-capped AgNP (synthesized by EDTA as reducing agent). The Ag concentration was adjusted to be identical for each sample. Among the tested samples, AgNPs@*Aloe vera* was shown to have the largest zone of inhibition around the discs, indicating the occurrence of significant antibacterial activity. Though, the mechanism of antibacterial activity of AgNP was not yet clearly understood, but the generalized mechanism is that the interaction of silver ion with the phosphorus moieties present in DNA of microorganism, resulting in the inactivation of DNA replication. The summary of antimicrobial activity of AgNP synthesized using *Aloe vera* plant extract and EDTA-capped AgNP synthesized by chemical reduction and *Aloe vera* plant extract are shown in Table 1. The inhibition studies were performed against different bacterial species including *Staphylococcus aureus*, *Bacillus cereus*, *Micrococcus luteus*, *Escherichia coli*, *Klebsiella pneumoniae*. These analyses against pathogenic bacteria have clearly displayed different inhibition rates in each case. Comparison between inhibitions by AgNP synthesized using *Aloe vera* plant extract and controls (only AgNP and only *Aloe vera* plant extract) revealed that there are two to four folds higher inhibition rate with the synthesized AgNP using *Aloe vera* plant extract. Comparisons among inhibitions are displayed in Additional file 1: Figure S1. The values were determined based on statistical standard deviation with the triplicate values. From these data, the AgNP using aloe vera extract (biological synthesis) show a significant activity compared to all the other samples.

**Table 1:** Antimicrobial activity of AgNP synthesized using *Aloe vera* plant extract and comparison of the antimicrobial activity of pure extract and AgNP

Microorganisms	*Aloe vera* extract	AgNP using *Aloe vera* extract (biological synthesis)	AgNP using chemical method (non-biological synthesis)	AgNO_3_
	Zone of inhibition (mm)			
*Staphylococcus aureus*	15	43	26	20
*Bacillus cereus*	13	38	22	18
*Micrococcus luteus*	19	33	13	15
*Escherichia coli*	26	42	14	22
*Klebsiella pneumoniae*	19	38	15	17

**Fig. 9:**
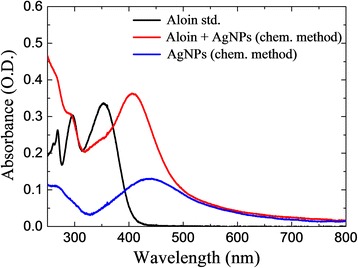
UV-visible spectra of AgNP synthesized by chemical method. Allowed reaction with an active molecule and the resulting spectral changes are shown

The UV-visible spectrum of the interacting behavior of the active component present in the *Aloe vera* with the freshly prepared AgNP was analyzed (Fig. 9). Figure 9 shows the UV-visible spectra of Aloin, Aloin-stabilized AgNP (biogenic method), and pure AgNP synthesized by chemical reduction method. For Aloin, the absorbance peaks appear at 268, 295, and 352 nm. The sharp peak appearing at 406 nm indicates the formation of AgNP stabilized by Aloin, and the broad peak appearing at 438 nm indicates the formation of pure AgNP synthesized by chemical reduction method. Then, the antibacterial activities against Aloin, AgNP-stabilized Aloin, and pure AgNP (chemical method) were compared and the zone of inhibition was calculated. The calculated standard deviations were between ±1 and ±2. Among these, AgNP stabilized aloin exhibits highest zone of inhibition values which indicates an enhanced antibacterial activity. Hence, these kinds of combined forms of herbal medicine can be used to treat the antibiotic-resistant bacterial strains.

The nano-sized AgNPs as antimicrobial agents have become more common as technological advances make their production more economical. One of the potential applications in which silver can be utilized is in management of plant diseases. Since silver displays multiple modes of inhibitory action to microorganisms, it may be used for controlling various plant pathogens in a relatively safer way compared to synthetic fungicides. The oxidation state of nanosilver strongly influences its ion leaching, and therefore, nanosilver exhibited much stronger antibacterial activity. Furthermore, the Ag^+^ ion releases about 5 and 60-nm silver particles, as well as macro-sized silver foil in the Ag-exposed surface area. So, antibacterial activity of nanosilver against gram positive and gram negative bacteria is better expressed by surface area, rather than mass or concentrations. Nano-scaled silver may exert these potentials: (1) releases silver ions and generates reactive oxygen species (ROS); (2) interacts with membrane proteins and influences their function; (3) accumulates in the cell membrane, affecting membrane permeability; and (4) enters into the cell where it can generate ROS, release silver ions, and affect DNA.

## Conclusions

Silver nanoparticles (AgNP) have been attested with the versatile preparation methods towards their potential applications in interdisciplinary sciences. With the current study, an additional strength has been given for the preparation using *Aloe vera* gel, as it is one of the important sources for the synthesis of spherical as well as octahedron-shaped AgNP. Methods were demonstrated by different synthetic methodologies (room temperature growth and microwave heating). The antimicrobial activities of the synthesized AgNP were tested against different Gram-negative and Gram-positive bacteria. The results claimed that the enhanced and synergetic activity of *Aloe vera* plant extract was obtained when it combined with AgNP. This study delineates an excellent SERS substrate with octahedron-shaped AgNP that can be synthesized by microwave heating using *Aloe vera* gel, which is one of the potential size and shape-directing agents.

## Additional file

Additional file 1: Figure S1. Comparison among inhibitions by AgNP (by chemical method), AgNP with Aloe vera plant extract, only *Aloe vera* plant extract and only AgNO3 solution. 1. *Staphylococcus aureus*; 2. *Bacillus cereus*; 3. *Micrococcus luteus*; 4. *Escherichia coli*; 5. *Klebsiella pneumoniae*. The values were determined based on statistical standard deviation with the triplicate values. The calculated standard deviations were between ±1 and ±2. (DOCX 84 kb)

## Abbreviations

4-ATP: 4-Aminothiophenol
AgNO_3_: Silver nitrate
AgNP: Silver nanoparticles
FE-SEM: Field-emission scanning electron microscopy
FTIR: Fourier transform infrared spectroscopy
GC-MS: Gas chromatography–mass spectrometry
HPLC: High-performance liquid chromatography
NaOH: Sodium hydroxide
PVP: Polyvinylpyrrolidone
SEM: Scanning electron microscopy
SERS: Surface-enhanced Raman spectroscopy/Raman Scattering
TEM: Transmission electron microscopy
XRD: X-ray powder diffraction
