# Supplementary material for: Shape- and Size-Controlled Synthesis of Silver Nanoparticles Using Aloe vera Plant Extract and Their Antimicrobial Activity
Source: Nanoscale Res Lett. 2016 Nov 25;11:520. doi: 10.1186/s11671-016-1725-x (PMC5122529; doi:10.1186/s11671-016-1725-x)
Supplement: Additional file 1: Figure S1. — Comparison among inhibitions by AgNP (by chemical method), AgNP with Aloe vera plant extract, only Aloe vera plant extract and only AgNO3 solution. 1. Staphylococcus aureus; 2. Bacillus cereus; 3. Micrococcus luteus; 4. Escherichia coli; 5. Klebsiella pneumoniae. The values were determined based on statistical standard deviation with the triplicate values. The calculated standard deviations were between ±1 and ±2. (DOCX 84 kb) [file 11671_2016_1725_MOESM1_ESM.docx]

**Shape and Size-controlled Synthesis of Silver Nanoparticles using *Aloe vera* Plant Extract and their Antimicrobial Activity**

Kaliyaperumal Logaranjan^1^, Anasdass Jaculin Raiza^1^, Subash C.B. Gopinath^2,3^, Yeng Chen^4,5^, Kannaiyan Pandian^1,^*

^1^Department of Inorganic Chemistry, University of Madras, Guindy Campus, Chennai, Tamil Nadu, India

^2^Institute of Nano Electronic Engineering, ^3^School of Bioprocess Engineering, University Malaysia Perlis, Kangar, Perlis, Malaysia

^4^Department of Oral & Craniofacial Sciences, ^5^Oral Cancer Research & Coordinating Center (OCRCC),  Faculty of Dentistry, University of Malaya, 50603 Kuala Lumpur, Malaysia

Figure S1. Comparison among inhibitions by AgNP (by Chemical method), AgNP with *Aloe vera* plant extract, only *Aloe vera* plant extract and only AgNO_3_ solution. 1. *Staphylococcus aureus*; 2. *Bacillus cereus*; 3. *Micrococcus ruteus*; 4. *Escherichia coli*; 5. *Klebsiella pneumonia*. The values were determined based on statistical standard deviation with the triplicate values. The calculated standard deviations were between ±1 and ±2.
